# *UvSorA* and *UvSorB* Involved in Sorbicillinoid Biosynthesis Contribute to Fungal Development, Stress Response and Phytotoxicity in *Ustilaginoidea virens*

**DOI:** 10.3390/ijms231911056

**Published:** 2022-09-21

**Authors:** Xuping Zhang, Dan Xu, Xuwen Hou, Penglin Wei, Jiajin Fu, Zhitong Zhao, Mingpeng Jing, Daowan Lai, Wenbing Yin, Ligang Zhou

**Affiliations:** 1 State Key Laboratory of Agrobiotechnology, Department of Plant Pathology, College of Plant Protection, China Agricultural University, Beijing 100193, China; 2 State Key Laboratory of Mycology, Institute of Microbiology, Chinese Academy of Sciences, Beijing 100101, China

**Keywords:** rice false smut disease, *Ustilaginoidea virens*, mycotoxins, sorbicillinoids, biosynthesis, *UvSorA*, *UvSorB*, gene deletion, phenotypes, biological functions

## Abstract

*Ustilaginoidea virens* (teleomorph: *Villosiclava virens*) is an important fungal pathogen that causes a devastating rice disease. It can produce mycotoxins including sorbicillinoids. The biosynthesis and biological functions of sorbicillinoids have not been reported in *U. virens*. In this study, we identified a sorbicillinoid biosynthetic gene cluster in which two polyketide synthase genes *UvSorA* and *UvSorB* were responsible for sorbicillinoid biosynthesis in *U. virens*. In ∆*UvSorA* and ∆*UvSorB* mutants, the mycelial growth, sporulation and hyphal hydrophobicity were increased dramatically, while the resistances to osmotic pressure, metal cations, and fungicides were reduced. Both phytotoxic activity of rice germinated seeds and cell wall integrity were also reduced. Furthermore, mycelia and cell walls of ∆*UvSorA* and ∆*UvSorB* mutants showed alterations of microscopic and submicroscopic structures. In addition, feeding experiment showed that sorbicillinoids could restore mycelial growth, sporulation, and cell wall integrity in ∆*UvSorA* and ∆*UvSorB* mutants. The results demonstrated that both *UvSorA* and *UvSorB* were responsible for sorbicillinoid biosynthesis in *U. virens*, and contributed to development (mycelial growth, sporulation, and cell wall integrity), stress responses, and phytotoxicity through sorbicillinoid mediation. It provides an insight into further investigation of biological functions and biosynthesis of sorbicillinoids.

## 1. Introduction

It is well known that fungi produce a diverse array of secondary metabolites (SMs) which have a variety of biological activities and functions. Some SMs are responsible for fungal growth and development, and some SMs act as armor and weaponry to help fungi to establish a secure niche in response to abiotic and biotic stresses [1,2,3,4,5]. Some pathogenic fungi usually produce SMs acting as virulence factors in pathogenicity, such as deoxynivalenol (DON) as a virulence factor for infection of wheat by *Fusarium graminearum* [6]. Up to now, the biological functions of most fungal SMs still remain unclear. Disruption, expression and overexpression of fungal SM biosynthesis related genes were considered as the practical strategies to elucidate their biological functions [7].

*Ustilaginoidea virens* (teleomorph: *Villosiclava virens*) is the fungal pathogen of rice false smut (RFS), one of the grain destructive diseases in the majority of rice-growing areas of the world. In particular, RFS has been estimated to occur in one third of the rice cultivation in China [8,9]. *U. virens* infects rice flowers and colonizes the inner flowers with mycelia, which are eventually transformed into false smut balls covered with yellow or dark green powdery chlamydospores [10]. In addition to its negative impacts on rice yield and quality, *U. virens* can produce poisonous mycotoxins to threaten the health of humans and animals [11,12,13] and can also inhibit the radicle and plumule growth of plant seedlings [14,15,16]. Up to now, three main types of mycotoxins, including 7 ustiloxins [15,17,18], 27 ustilaginoidins [14,19,20,21,22], and 21 sorbicillinoids [16,23] have been identified in *U. virens*. These mycotoxins were screened to show cytotoxic, antimicrobial and phytotoxic activities [15,16,23].

Sorbicillinoids are a family of polyketide mycotoxins that are related to the hexaketide sorbicillin, and typically contain a sorbyl side chain in their structures [24]. To date, at least 159 sorbicillinoids have been identified, and they were found in both terrestrial and marine fungi [24,25,26]. The studies of sorbicillinoids have been currently focused on their structural identification [24,25,26], biological activities including radical-scavenging activity [27] and cytotoxic activity [23,28], and biosynthetic pathways [29], as well as the enzymes related to the sorbicillinoid biosynthesis [30,31]. To the best of our knowledge, the biological functions of sorbicillinoids in *U. virens* are rarely known. In this study, we identified a sorbicillinoid biosynthetic gene cluster (BGC) which contained two key structural genes *UvSorA* and *UvSorB* in *U. virens*. Both *UvSorA* and *UvSorB* belonged to highly reducing polyketide synthase (HR-PKS) gene and non-reducing polyketide synthase (NR-PKS) gene, respectively. To further study the biological functions of these two genes related to sorbicillinoid biosynthesis, we generated two deletion mutants namely Δ*UvSorA* and Δ*UvSorB*, as well as their complemented strains. Both UvSorA and UvSorB were identified as the PKSs that were essential for sorbicillinoid biosynthesis. They contributed to development (i.e., mycelial growth, sporulation, and cell wall integrity), stress response, and phytotoxicity through sorbicillinoid mediation in *U. virens*.

## 2. Results

### 2.1. Identification and Characteriation of UvSorA and UvSorB in U. virens

#### 2.1.1. Identification of the Sorbicillinoid Biosynthesis Gene Cluster in *U. virens*

**Figure 1:**
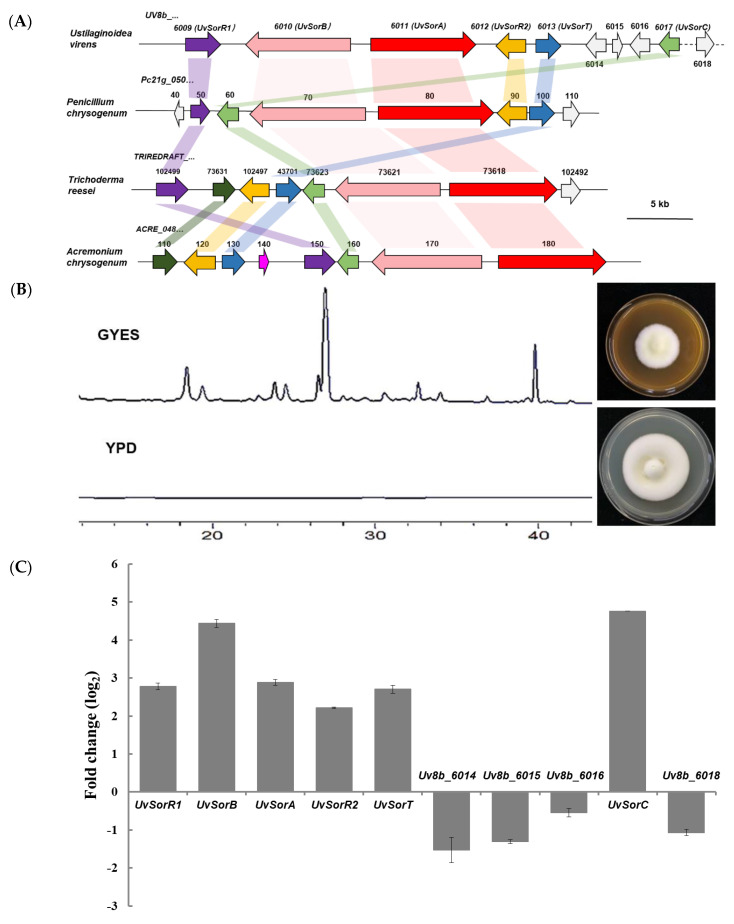

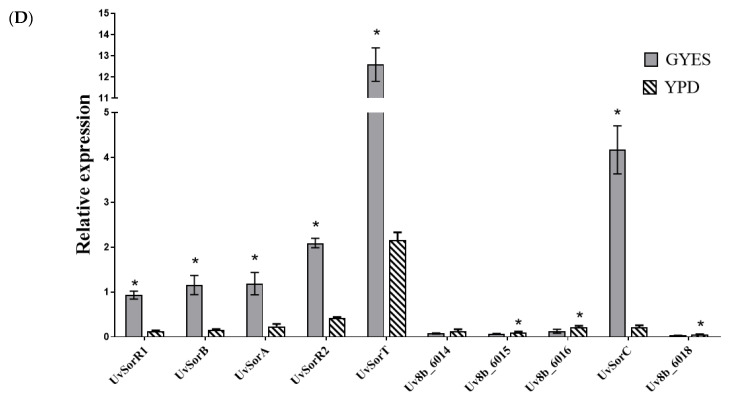
Identification of the *sor* BGC in *U. virens*. (**A**) Comparison of *sor* BGCs among *U. virens, P. chrysogenum, T. reesei,* and *A. chrysogenum.* Orthogous genes were presented with the same colors; (**B**) HPLC profiles of the methanol extracts of the mycelia cultured on the sorbicillinoids-producing medium (GYES) and sorbicillinoids-non-producing medium (YPD) detected at 370 nm (left panel). The peaks were identified as sorbicillinoids by LC−MS. Colony morphologies (front view) observed after 28 days of culture on the GYES and YPD plates (right panel); (**C**) The expression pattern of *sor* BGC genes analyzed by RNA−Seq. The fold change was equal to averaged FPKM of *U. virens* cultured in GYES/YPD; (**D**) The qRT−PCR expression level of *sor* BGC genes in *U. virens* cultured in GYES compared to YPD medium. The values represent the average ± SD of three biological replicates. The asterisk (*) represents a significant difference at *p* < 0.05.

The potential BGCs for SMs in *U. virens* were analyzed by the antiSMASH (antibiotics & secondary metabolite analysis shell) [32]. A total of 19 gene clusters for SMs were identified, which included five type I PKS clusters. A putative gene cluster located on chromosome 2 was predicted to be involved in the biosynthesis of sorbicillinoids with 71% similarity (Table S1). This putative sorbicillinoid BGC (*sor* BGC) was predicted to contain 10 genes from *UV8b_6009* to *UV8b_6018* that were located in an about 40 kb region [33]. By blastP searches for orthologous genes in the NCBI (National Center for Biotechnology Information), six genes *UV8b_6011* (*UvSorA*), *UV8b_6010* (*UvSorB*), *UV8b_6009* (*UvSorR1*), *UV8b_6012* (*UvSorR2*), *UV8b_6013* (*UvSorT*) and *UV8b_6017* (*UvSorC*) were found to be conserved in *U. virens*, *Penicillium chrysogenum*, *Trichoderma reesei*, and *Acremonium chrysogenum* (Figure 1A). Among these conserved genes, two PKS genes *UvSorA* and *UvSorB* showed high homology with its orthologues in other fungi. *UvSorA* showed amino acid identities of 70% with *Pc21g_05080*, 72% with *TRIREDRAFT_73618*, and 71% with *ACRE_048180*, respectively. Another putative PKS gene *UvSorB* shared 65%, 64% and 65% amino acid similarity with *Pc21g_05070*, *TRIREDRAFT_73621*, and *ACRE_048170*, respectively (Table S2).

Composition changes in the medium could affect general metabolic profile of an organism, based on the approach of one strain-many compounds (OSMAC) [34]. It was found that sorbicillinoids were produced when *U. virens* was cultured in GYES medium and were not produced in YPD medium (Figure 1B). This result provided clues to identify *sor* BGC using sorbicillinoids-producing vs. non-producing media. RNA−Seq approach and qRT−PCR analysis were pursued to analyze the expression patterns of genes in *sor* BGC when *U. virens* was cultured in two types of media for 10 days. RNA−Seq data analysis showed that, except for *UV8b_6014*, *UV8b_6015*, *UV8b_6016*, and *UV8b_6018*, the expression levels of six conserved genes in putative *sor* BGC were upregulated significantly when *U. virens* was cultured in GYES compared to YPD medium (Figure 1C). Two structural genes (also known as backbone genes) *UvSorA* and *UvSorB* were upregulated 2.9−fold and 4.4−fold (log_2_FC), respectively (Table S3). In addition, qRT−PCR analysis also showed the same expression pattern with RNA−Seq (Figure 1D). Therefore, a gene cluster, which was named as *sor* BGC, was identified in *U. virens*.

#### 2.1.2. *UvSorA* and *UvSorB* as Polyketide Synthase Genes of Sorbicillinoid Biosynthesis

**Figure 2:**
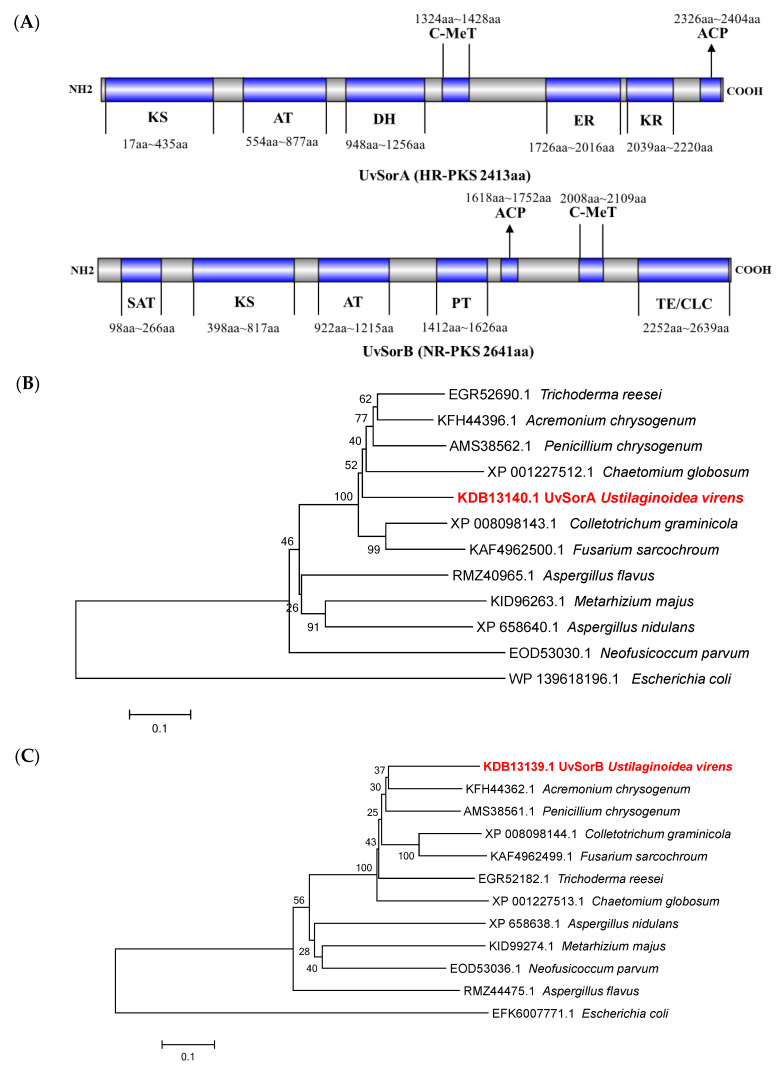
Identification and characterization of UvSorA and UvSorB. (**A**) Schematic diagram of UvSorA and UvSorB functional domains analysis. ACP, acyl carrier protein; AT, acyl transferase; C-MeT, C-methyltransferase; DH, dehydratase; ER, enoylreductase; KR, β-ketoacyl reductase; KS, β-ketoacyl synthase; PT, product template; SAT, starter unit ACP transacylase; TE/CLC, thioesterase/Claisen cyclase. (**B**) Phylogenetic analysis of UvSorA with different orthologous. (**C**) Phylogenetic analysis of UvSorB with different orthologous. The phylogenetic tree was constructed using the neighbor-joining (NJ) method in MEGA5.0.

Two PKSs UvSorA and UvSorB were predicted as iterative type I polyketide synthases. *UvSorA* consisted of a predicted open reading frame (ORF) with 7591 bp interrupted by five introns and encoded a protein with 2413 amino acids. UvSorA was deduced as an HR−PKS, which contained seven characteristic structural domains, involved in the functions of β-ketoacyl synthase (KS), acyl transferase (AT), dehydratase (DH), C-methyltransferase (C-MeT), enoylreductase (ER), β-ketoacyl reductase (KR), and acyl carrier protein (ACP) (Figure 2A). *UvSorB* consisted of a predicted open reading frame with 8058 bp interrupted by two introns and encoded an NR-PKS with 2641 amino acids. It also included KS, AT, ACP, and C-MeT domains. In addition, UvSorB embodied other domains like starter unit ACP transacylase (SAT), product template (PT), and thioesterase/Claisen cyclase (TE/CLC) (Figure 2A). Amino acid sequence alignment analysis revealed that the functional domains of UvSorA and UvSorB were well conserved among *P. chrysogenum*, *T. reesei*, *A. chrysogenum* and *Colletotrichum graminicola* (Figure S1).

Two phylogenetic trees were constructed based on *UvSorA* and *UvSorB* orthologues of *U. virens* with other fungi which contained *sor* BGC, with *Escherichia coli* as the outgroup. The phylogenetic tree revealed that UvSorA protein showed a high similarity to KFH44396.1 from *A. chrysogenum* (Figure 2B). Similarly, the UvSorB protein was the most similar to KFH44362.1 from *A. chrysogenum* (Figure 2C). All these results showed that both *UvSorA* and *UvSorB* from *U. virens* had a close relationship with two PKS genes in *sor* BGC of *A. chrysogenum*, and were specifically conserved in *P. chrysogenum*, *T. reesei* and *C. globosum*.

In order to confirm the functions of *UvSorA* and *UvSorB*, we deleted the coding region of *UvSorA* or *UvSorB* by replacing it with a geneticin-resistance (*NeoR*) cassette employing a previously described transformation method [35]. The deletion fragments flanking approximately 1.0 kb upstream and downstream of *UvSorA* or *UvSorB* ORF regions were fused partially with the geneticin-resistance gene. The gRNA spacers of *UvSorA* and *UvSorB* were cloned into the CRISPR-Cas9 vector (pCas9-tRp-gRNA). Both Δ*UvSorA* and Δ*UvSorB* mutants were generated by replacing the endogenous *UvSorA* or *UvSorB* ORF with the deletion cassette via the protoplast transformation with linear donor DNA fragment and the CRISPR construct.

Genomic DNA was extracted from the transformants, and correct gene replacements were confirmed by diagnostic PCR [36]. PCR assays using specific pairs of primers (Table S5) for the deletion of *UvSorA* or *UvSorB* and presence of *NeoR* in transformants, and then further verified homologous recombination in both upstream and downstream flanking sequences.

**Figure 3:**
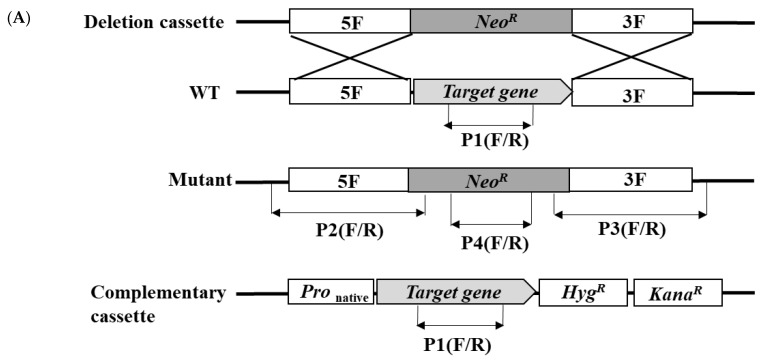

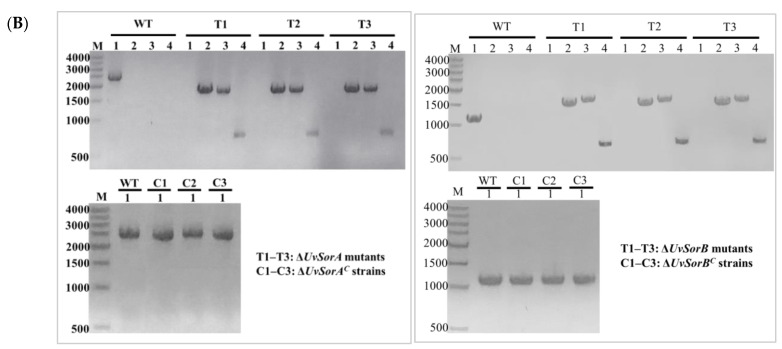
Generation of ∆*UvSorA* and ∆*UvSorB* mutants and their complemented strains of *U. virens*. (**A**) Schematic illustration of disruption and complementation of the target gene (*UvSorA* or *UvSorB*). (**B**) In the Δ*UvSorA* mutant, the specific bands (1800, 1772 and 687 bp) were only detected in the mutants (T1–T3) but not in WT strain. The specific band (2387 bp) was detected in Δ*UvSorA^C^* strains (C1–C3) (left panel); In the Δ*UvSorB* mutants, the specific bands (1532, 1634 and 687 bp) were only detected in the mutants (T1–T3) but not in WT strain. The specific band (1172 bp) was detected in Δ*UvSorB^C^* strains (C1–C3) (right panel). Lane M: 500 bp DNA Ladder.

To determine whether the altered phenotype in ∆*UvSorA* or ∆*UvSorB* mutant could be restored by re-introduction of a wild-type copy of *UvSorA* or *UvSorB*, the ORF sequence of *UvSorA* or *UvSorB* containing native promoter was introduced into the deletion mutant with complementation vector (pCBHT) to create the complemented strain Δ*UvSorA*^C^ or Δ*UvSorB^C^*, respectively. The complemented transformants were confirmed by diagnostic PCR using designated primers (Table S5). Three of the deletion mutants (T1, T2 and T3) and three of complemented strains (C1, C2 and C3) were selected for the subsequent phenotype test (Figure 3).

**Figure 4:**
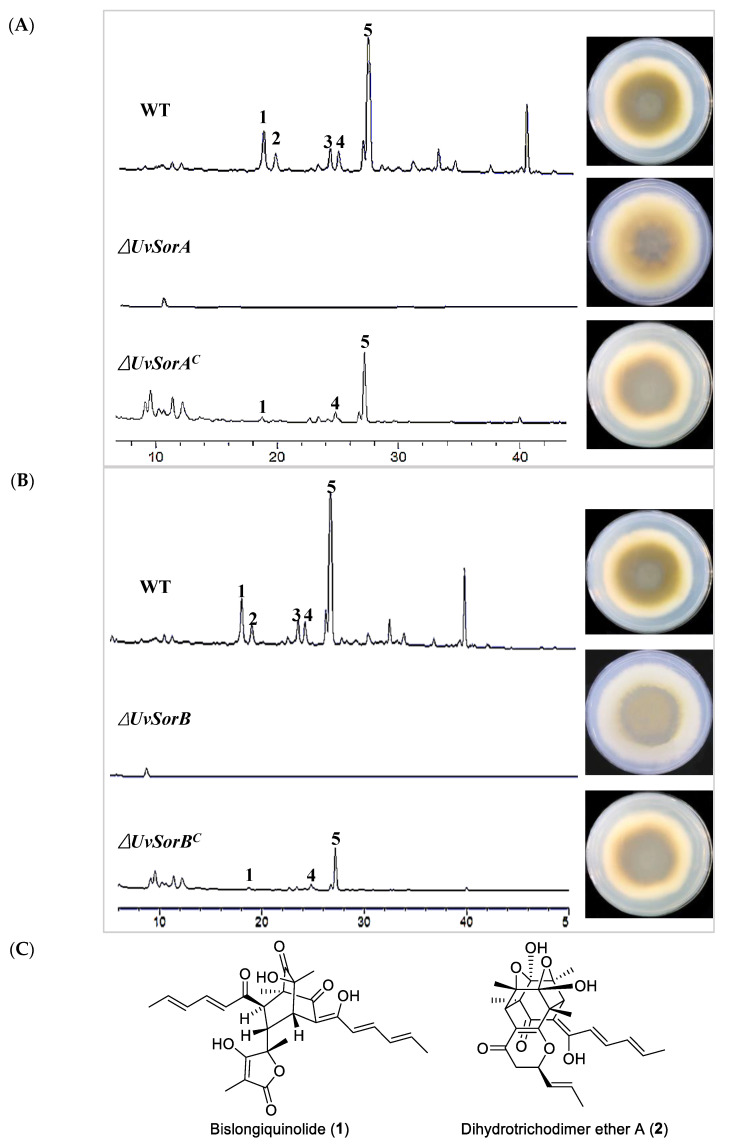

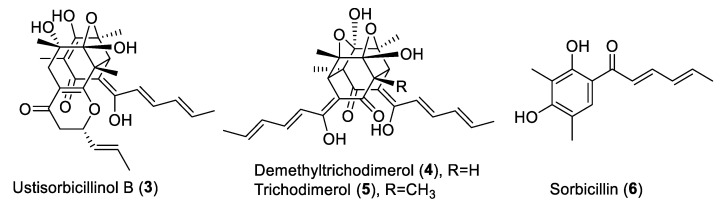
Colony morphologies and HPLC profiles of sorbicillinoids from WT strain, deletion mutants and complemented strains of *U. virens.* (**A**) HPLC profiles of the WT strain, Δ*UvSorA* mutant and Δ*UvSorA*^C^ strain detected with UV at 370 nm (left panel). Colony morphologies (back view) observed after 28 days of culture on the PDA plates (right panel); (**B**) HPLC profiles of the WT strain, Δ*UvSorB* mutant and Δ*UvSorB^C^* strain detected with UV at 370 nm (left panel). Colony morphologies (back view) observed after 28 days of culture on the PDA plates (right panel); (**C**) Chemical structures of sorbicillinoids (**1**)–(**6**).

To verify whether *UvSorA* and *UvSorB* were responsible for the biosynthesis of sorbicillinoids, the wild-type (WT) strain, deletion mutants and complemented strains were all cultured in GYES medium at static condition, and sorbicillinoids were extracted with methanol (MeOH) from the filtered hyphae for HPLC analysis. Five main peaks (compounds **1**–**5**) in WT strain were identified as trichotetronine (**1**), dihydrotrichodimer ether A (**2**), ustisorbicillinol B (**3**), demethyltrichodimerol (**4**), trichodimerol (**5**) by HPLC comparing with authentic compounds (Figure 4A,B), and LC−MS with their quasi-molecular negative-ion peaks at *m/z* 423.1187, 495.2036, 497.2195, 481.1863, and 495.2018 [M−H]^-^ in the HR-ESI-MS, respectively [16]. Their structures are shown in Figure 4C. Neither sorbicillinoid derivatives nor their biosynthetic intermediates were detectable in ∆*UvSorA* and ∆*UvSorB* mutants. From the back views of the colonies shown in the right panels of Figure 4A,B, more pigments were secreted into the PDA medium from WT strains than those from the deletion mutants. In addition, the biosynthesis of sorbicillinoids was partially restored in ∆*UvSorA^C^* or ∆*UvSorB^C^* strain by gene complementation (Figure 4A,B).

**Figure 5:**
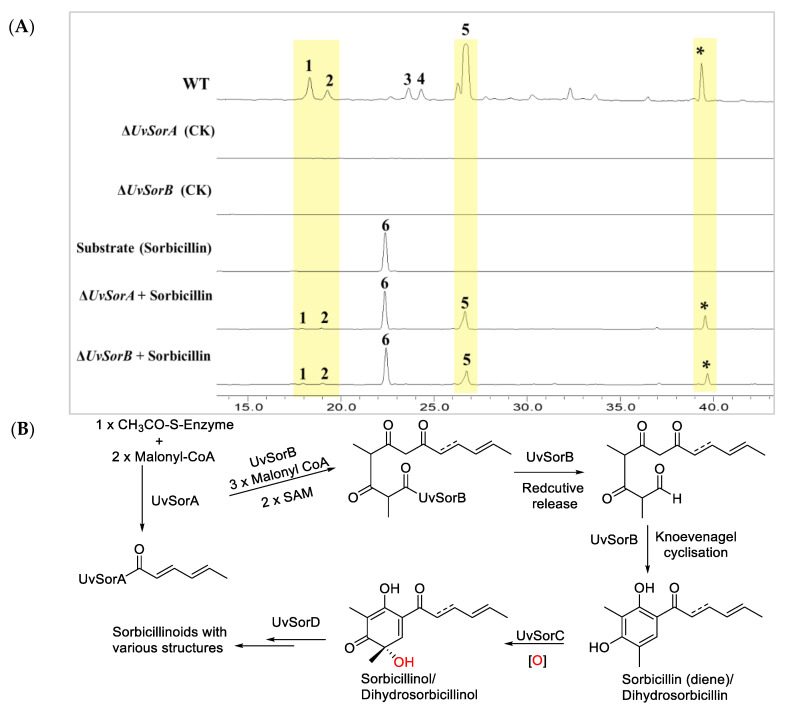
Feeding experiments and proposed sorbicillinol biosynthetic pathway. (**A**) HPLC profiles of WT strain, ∆*UvSorA* and ∆*UvSorB* mutants, and deletion mutants fed with sorbicillin. The peak marked with an asterisk (*) was presumed as an unidentified sorbicillinoid according to its UV spectrum; (**B**) Summary of the proposed sorbicillinoid biosynthetic pathway in *U. virens*.

Precursor feeding was considered to restore the abolished metabolite production in the deletion mutant [2], so sorbicillin (**6**) was fed to the deletion mutant Δ*UvSorA* or Δ*UvSorB*, and it was found that either Δ*UvSorA* or Δ*UvSorB* mutant could restore production of sorbicillinoids (Figure 5A).

Gene deletion, complementation, and precursor feeding experiments provided direct evidence that *UvSorA* and *UvSorB* as the structural genes catalyzed the earlier steps of sorbicillinoid biosynthesis, in which acetyl-CoA and malonyl-CoA were cyclized to form the primary polyketides (PKs) sorbicillin (**6**) and dihydrosorbicillin. Summary of the proposed sorbicillinoid biosynthetic pathway in *U. virens* is shown in Figure 5B by referencing the biosynthetic pathway of *Trichoderma reesei* QM6a [29].

### 2.2. Deletion of UvSorA and UvSorB Increased Mycelial Growth, Sporulation and Hyphal Hydrophobicity

#### 2.2.1. Deletion of *UvSorA* and *UvSorB* Increased Mycelial Growth and Sporulation

**Figure 6:**
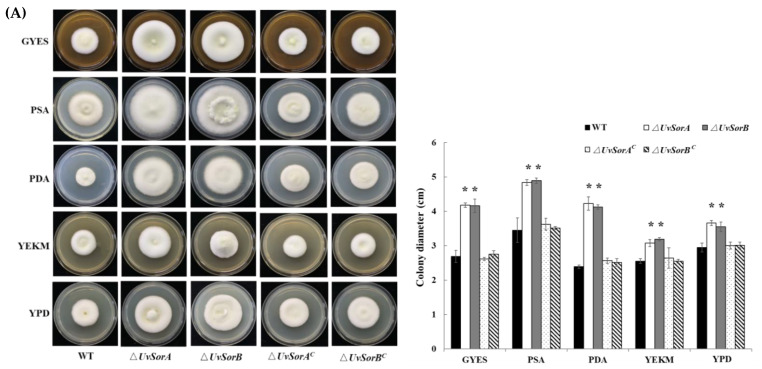

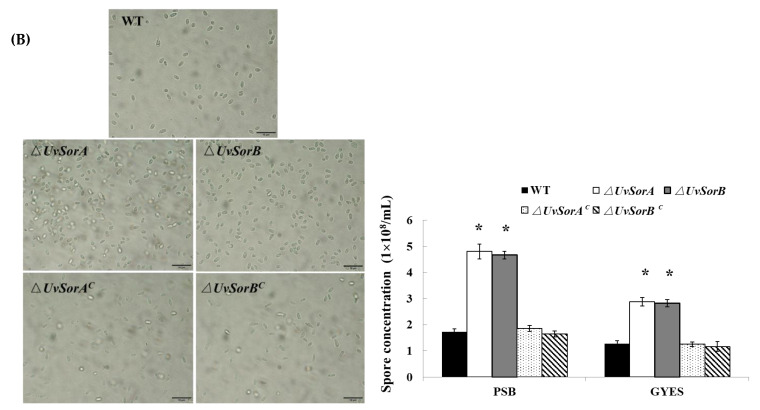
*UvSorA* and *UvSorB* disruption resulted in an increased mycelial growth and sporulation. (**A**) Mycelia growth of the tested strains cultured at 28 °C for 18 days on different media (left panel). Statistical analysis of the colony diameters (right panel); (**B**) Microscope detection of the spores collected from the tested strains cultured in PSB medium at 28 °C with 160 rpm for 7 days. Scale bar = 20 μm (left panel). Statistical analysis of spore concentrations in PSB medium and GYES medium (right panel). Three independent biological experiments were performed with three replicates each time, with similar results yielded in each biological experiment; Error bars represent the standard deviation, and asterisk (*) represents a significant difference at *p* < 0.05.

Abolishment or overproduction of specific SMs can alter fungal development, such as mycelial growth and sporulation [2,4]. In order to investigate the functions of *UvSorA* and *UvSorB* genes and sorbicillinoids in *U. virens* mycelial growth and sporulation, we cultured WT strains, deletion mutants and complemented strains on GYES, PSA, PDA, YEKM and YPD media. The Δ*UvSorA* and Δ*UvSorB* mutants both showed a significantly fast growth on all tested media (Figure 6A). The dry weight of hyphae of Δ*UvSorA* and Δ*UvSorB* mutants was also higher than WT and complemented strains cultured in liquid PSB and GYES media (Figure S2). The sporulation increased approximately 2.5-fold in mutants compared with the WT and complemented strains (Figure 6B). The results showed that both hyphal growth and sporulation increased in ∆*UvSorA* and ∆*UvSorB* mutants.

**Figure 7:**
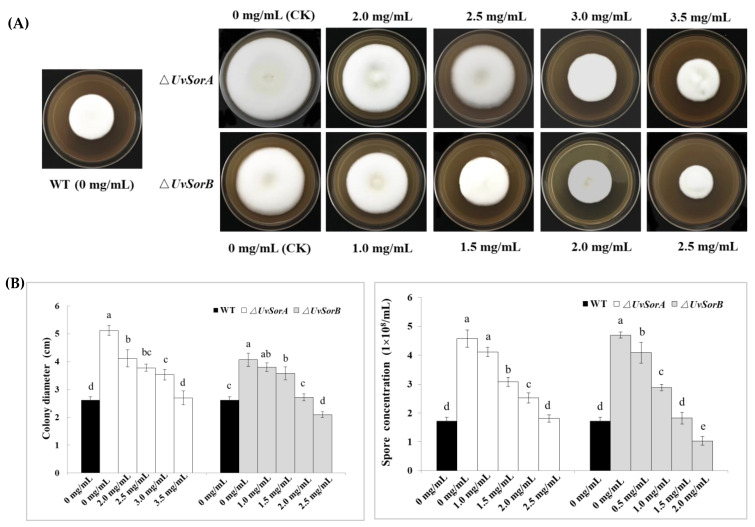
Sorbicillinoids feeding experiment in ∆*UvSorA* and ∆*UvSorB* mutants. (**A**) Colony morphology of Δ*UvSorA* and Δ*UvSorB* mutants were incubated on GYES at 28 °C for 21 days, supplemented with different concentrations of sorbicillinoids; (**B**) Statistical analysis of colony diameters (left panel) and spore concentrations (right panel) in feeding experiment. *ΔUvSorA* and *ΔUvSorB* mutants and WT were incubated on PSB at 28 °C with 160 rpm for 7 days supplemented with different concentrations of sorbicillinoids. Three independent biological experiments were performed with three replicates each time. Error bars represent the standard deviation. Different letters (a–e) indicate significant differences among the data of each treatment at *p* < 0.05.

Both Δ*UvSorA* and Δ*UvSorB* mutants completely lost ability to produce sorbicillinoids. To verify mycelial growth and sporulation were directly mediated by sorbicillinoids, we fed different concentrations (1.0–3.5 mg/mL) of total sorbicillinoids to the ∆*UvSorA* and ∆*UvSorB* mutants (Figure 7A). The mycelial growth rate of the deletion mutants was found to be restored to the WT when the sorbicillinoids with concentration of 3.5 mg/mL for ∆*UvSorA* and 2.0 mg/mL for ∆*UvSorB* were added (Figure 7B). In addition, feeding sorbicillinoids also could restore the spore production level of ∆*UvSorA* and ∆*UvSorB* mutants. The spore production level could be completely restored to the level of WT with sorbicillinoids concentration at 2.5 mg/mL for ∆*UvSorA*, and 1.5 mg/mL for ∆*UvSorB* (Figure 7B). These results indicated that both *UvSorA* and *UvSorB* might play negative regulatory roles in hyphal growth and sporulation through sorbicillinoid mediation.

#### 2.2.2. Deletion of *UvSorA* and *UvSorB* Increased Hyphal Hydrophobicity

**Figure 8:**
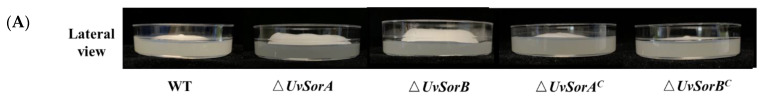

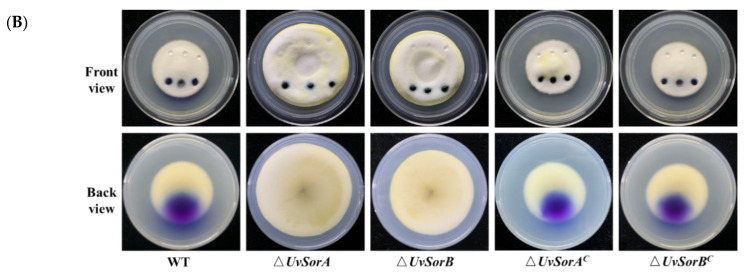
Effects of ∆*UvSorA* and ∆*UvSorB* mutants on aerial hyphae growth and hydrophobicity. (**A**) Aerial hyphae of ∆*UvSorA* and ∆*UvSorB* mutants grew faster than WT and complemented strains. All tested strains were cultured on PSA medium for 18 days at 28 °C; (**B**) Spherical water and bromophenol blue solution droplets (20 μL for each drop) were placed on colonies of *∆UvSorA* and *∆UvSorB* mutants, whereas the droplet dispersed on the colony of WT and complemented strains.

The hydrophobic property on cell surface is a distinct feature of aerial hyphae in many fungal species [37]. Both ∆*UvSorA* and ∆*UvSorB* mutants presented as the raised colonies due to the increased aerial hyphal growth, suggesting that each mutant might have an increased hydrophobicity on hyphal surface (Figure 8A). To confirm this deduction, 20 μL (three replications) of 2.5% bromophenol blue solution or 20 μL (three replications) of ddH_2_O was placed on each colony surface of the tested strains grown on PSA medium. As shown in Figure 8B, due to more aerial hyphae were present, both 2.5% bromophenol blue solution and ddH_2_O maintained spherical droplets on the surface of Δ*UvSorA* and Δ*UvSorB* colonies without being absorbed or extended for more than 12 h, thereby demonstrating the strong hydrophobicity of the Δ*UvSorA* and Δ*UvSorB* hyphae. These results indicated that the *UvSorA* and *UvSorB* were involved in the growth and hydrophobicity of aerial mycelia in *U. virens*.

### 2.3. Deletion of UvSorA and UvSorB Decreased Resistances to Osmotic, Metal Cation and Fungicide Stresses

#### 2.3.1. Deletion of *UvSorA* and *UvSorB* Resulted in Defects in Response to Hyperosmotic and Metal Cation Stresses

**Figure 9:**
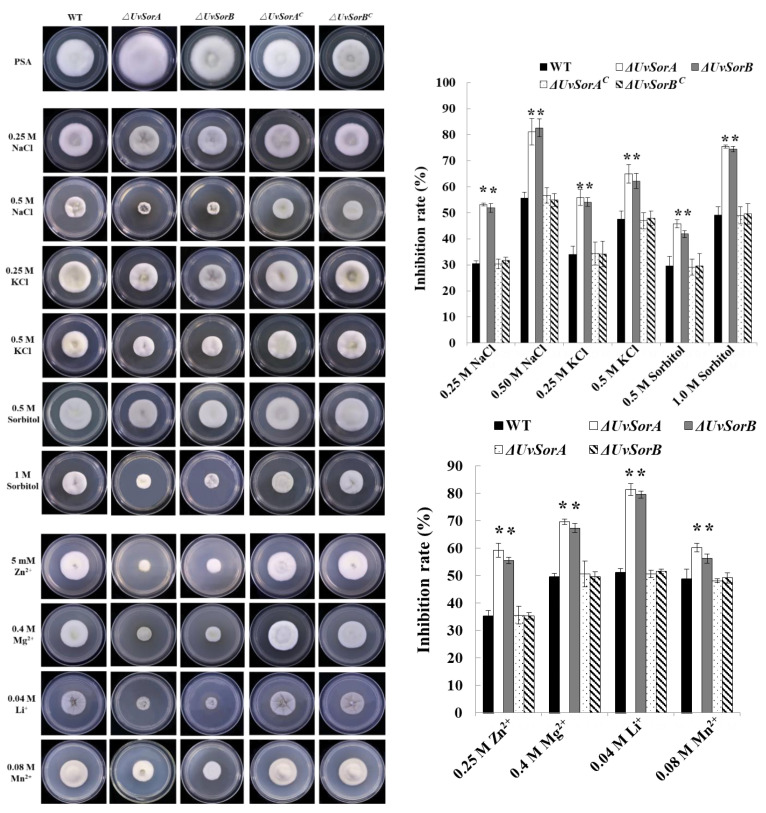
*UvSorA* and *UvSorB* contributed to the tolerance to hyperosmotic and metal cation stresses. The colony growth of mutants under hyperosmotic and metal cations stresses grown at 28 °C for 18 days (left panel); Statistical analysis of mycelial growth inhibition rate under hyperosmotic (upper right panel) and metal cations stresses (lower right panel). Measurements of growth inhibition rate of each treated strain are relative to that of each untreated control. Three independent biological experiments were performed with three replicates each time. Error bars represent the standard deviation, and the asterisk (*) represents a significant difference at *p* < 0.05, ** *p* < 0.01).

Biosynthesis of SMs is usually activated when the fungus responses to abiotic and biotic stresses [38]. The classical method for activating secondary metabolism is the manipulation of culture conditions, such as addition of metal ions [39,40,41]. To investigate whether *UvSorA* and *UvSorB* were included in response to hyperosmotic and metal cation stresses, we assayed the defects of the knockout mutants, complemented and WT strains cultured on PSA or PSA amended with 0.25 or 0.5 M NaCl, 0.25 or 0.5 M KCl, 0.5 or 1.0 M sorbitol, 5 mM Zn^2+^, 0.4 M Mg^2+^, 0.04 M Li^+^, 0.08 M Mn^2+^ (left panel of Figure 9). The mycelial growth inhibition rate of ∆*UvSorA* and ∆*UvSorB* mutants under either hyperosmotic or metal cation stresses increased 1.2–1.7 folds compared with the WT and complemented strains (right panel of Figure 9). These results indicated that *UvSorA* and *UvSorB* played a positive role in hyperosmotic and metal cation responses in *U. virens*.

#### 2.3.2. Deletion of *UvSorA* and *UvSorB* Increased Sensitivity to Fungicides

**Figure 10:**
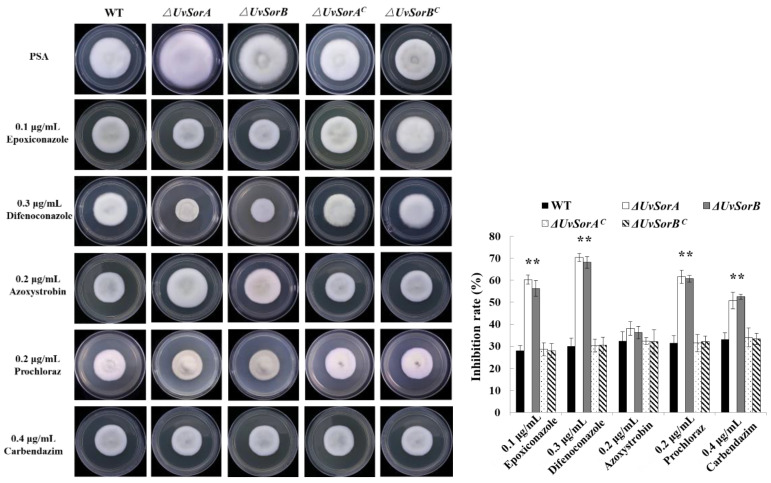
Effects of fungicides on mycelia growth of ∆*UvSorA* and ∆*UvSorB* mutants. The colony growth of the strains under fungicide stresses (left panel). Statistical analysis of mycelial growth of the strains under fungicide stresses (right panel). Each strain was incubated on PSA supplemented with a certain fungicide at 28 °C for 18 days. Three independent biological experiments were performed with three replicates each time. Error bars represent the standard deviation, and the asterisk (*) represents a significant difference at *p* < 0.05, ** *p* < 0.01.

Plant disease control strategies include genetic resistance, chemical and biological control, and cultivation practices. Currently, RFS disease control largely relied on fungicides, such as propiconazole, azoxystrobin difenoconazole, prochloraz and carbendazim [42,43]. Carbendazim interfered with spindle formation during mitosis and affected cell division [44]. Epoxiconazole, difenoconazole and prochloraz disturbed the formation of cell walls through ergosterol synthesis inhibition in fungi [45]. Azoxystrobin acted as a mitochondrial respiratory inhibitor, it potently inhibited respiration of wheat pathogen *Septoria tritici* at the level of Complex III [46]. Considering Δ*UvSorA* and Δ*UvSorB* mutants had deficiency in response to hyperosmotic and metal cation stresses, we suspected that Δ*UvSorA* and Δ*UvSorB* mutants might be more sensitive to fungicides. So, the sensitivity of tested strains to five fungicides was analyzed. The strains were cultured on PSA or added with 0.1 μg/mL epoxiconazole, 0.3 μg/mL difenoconazole, 0.2 μg/mL azoxystrobin, 0.2 μg/mL prochloraz, and 0.4 μg/mL carbendazim, respectively. The fungicide inhibition rates of the Δ*UvSorA* and Δ*UvSorB* mutants were 1.5 times greater than those of WT and complemented strains. These results indicated that both ∆*UvSorA* and ∆*UvSorB* mutants increased sensitivity to four tested fungicides except azoxystrobin (Figure 10).

### 2.4. Culture Filtrates of ∆UvSorA and ∆UvSorB Showed a Decreased Inhibition on Germ Elongation of Rice Germinated Seeds

**Figure 11:**
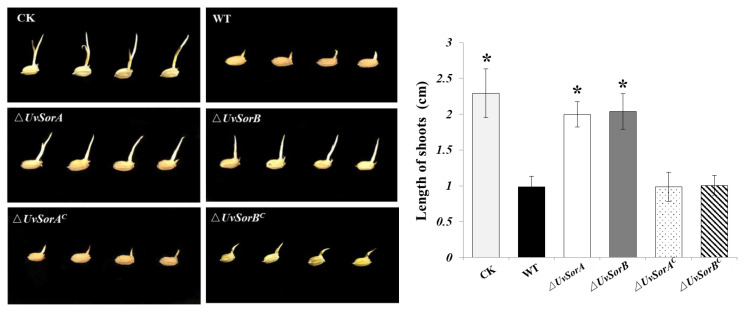
The growth of rice germinated seeds treated with testing culture filtrates. The rice seeds were soaked in 25 mL culture filtrates of tested strains in 100 mL-conical flasks at 28 °C for 7 days (**left pane**l); Statistical analysis of germinated rice seeds (**right panel**). The length of shoots was measured after the incubation. Three independent biological experiments were performed with three replicates each time. Error bars represent the standard deviation, and the asterisk (*) represents a significant difference at *p* < 0.05.

Several studies have demonstrated that some SMs involved in the pathogenesis as virulence factors and were able to modify the physiological functions of the host cells [47,48,49]. Sorbicillinoids as one group of mycotoxins produced by *U. virens* might play important roles in fungus–host interaction. The culture filtrates separated from 14-day-old PDB cultures of WT, deletion mutants and complemented strains were used to soak rice seeds to evaluate the role of sorbicillinoids in the inhibition on germ elongation of rice germinated seeds. Shoot growth was examined after incubation at 28 °C for 7 days. The germ elongation of rice seeds was significantly decreased in the samples treated with culture filtrates of WT and complemented strains, whereas the germ elongation of rice seeds treated with the culture filtrates of ∆*UvSorA* or ∆*UvSorB* mutants was close to that of the untreated rice seeds (Figure 11). This result showed that *UvSorA* and *UvSorB* involved in sorbicillinoids production would be toxic to rice seeds. Both ∆*UvSorA* and ∆*UvSorB* decreased inhibition on germ elongation of rice germinated seeds which indicated that biosynthesized sorbicillinoids had an inhibitory activity on germ elongation of rice germinated seeds. It was verified by the previous in vitro results about the inhibition of sorbicillioids on seed germination of rice and lettuce [16].

### 2.5. Deletion of UvSorA and UvSorB Resulted in Deficiency of Cell Wall Integrity

#### 2.5.1. Deletion of *UvSorA* and *UvSorB* Resulted in Decrease of Tolerance to Cell Wall Damaging Agents

**Figure 12:**
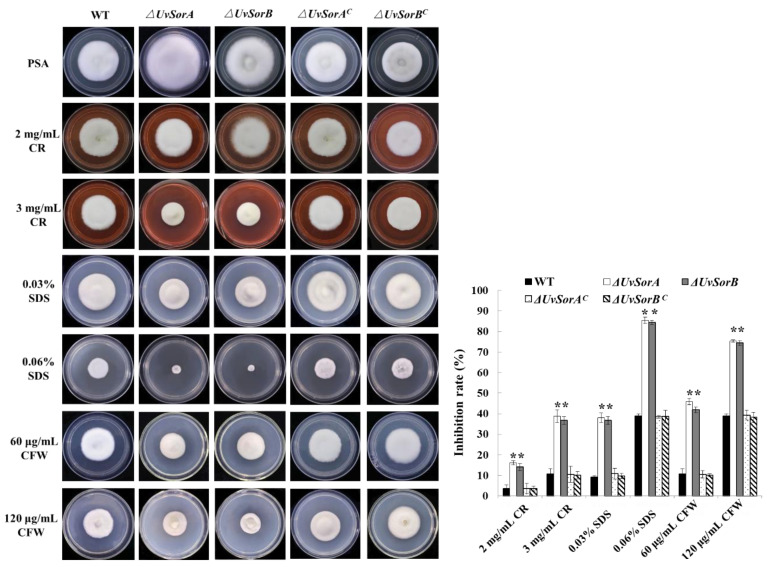
*UvSorA* and *UvSorB* contributed to the tolerance to cell wall damaging agents. The colony of tested strains under the stresses of cell wall damaging agents grown at 28 °C for 18 days (**left panel**). Statistical analysis of mycelial growth inhibition rate under cell wall stresses (**right panel**). Three independent biological experiments were performed with three replicates each time. Error bars represent the standard deviation, and the asterisk (*) represents a significant difference at *p*< 0.05, ** *p* < 0.01.

The effects of the fungal secondary metabolites on cell wall impairment have been broadly studied and suggested that the production of some SMs can be induced in response to cell wall stress [50,51,52]. Deletion of *UvSorA* and *UvSorB* led to decreases in resistance to hyperosmotic, metal cation and fungicide stresses (Figure 9 and Figure 10), which indicated that *UvSorA* and *UvSorB* might regulate the integrity of the cell wall through sorbicillinoid mediation. To prove this hypothesis, we tested the sensitivity of Δ*UvSorA* and Δ*UvSorB* mutants to cell wall damaging agents. The mycelia of tested strains were cultured on PSA or PSA amended with 2 or 3 mg/mL Congo red (CR), 0.03 or 0.06% dodecyl sulfate (SDS), 60 or 120 μg/mL calcofluor white (CFW). It appeared that the inhibition rate of mycelial growth increased significantly (2.0–4.5 folds) on both ∆*UvSorA* and *∆UvSorB* mutants compared with the WT and complemented strains, indicating that the deletion of *UvSorA* and *UvSorB* could decrease the tolerance of *U. virens* to cell wall damaging agents (Figure 12).

#### 2.5.2. Deletion of *UvSorA* and *UvSorB* Resulted in Alterations of Microscopic and Submicroscopic Structures of Mycelia and Cell Walls

**Figure 13:**
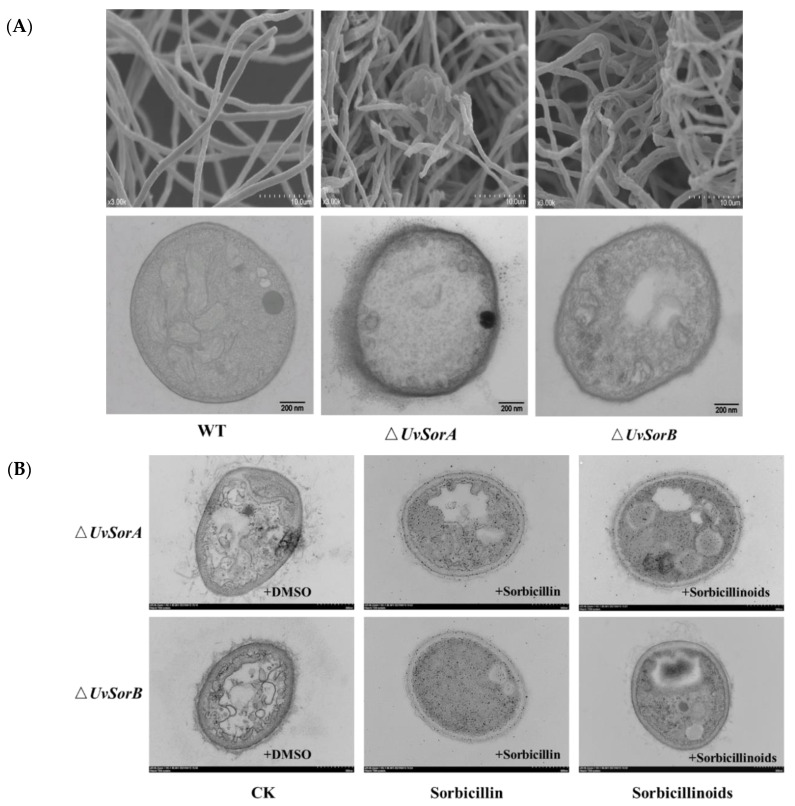
Microscopic and submicroscopic alterations of mycelia structures observed and analyzed by SEM and TEM. (**A**) The WT, Δ*UvSorA*, and Δ*UvSorB* mutants were grown on PSA for 10 days at 28 °C. Up column: scale bar = 10 µm, down column: scale bar = 200 nm; (**B**) Ultrastructural analyses of the cell wall integrity of feeding experiment. Sorbicillin and crude sorbicillinoid extract were dissolved in 10% DMSO, scale bar = 500 nm.

Deletion of *UvSorA* and *UvSorB* led to a decrease in resistance to osmotic, metal cations, cell wall damaging agents and fungicides, which indicated that the microscopic and submicroscopic structures of mycelia and cell wall might be altered. To prove it, we observed the alterations of morphology, including mycelia and cell wall by scanning electron microscopy (SEM) and transmission electron microscope (TEM). Analysis of mycelium SEM images showed that the WT strain exhibited normal morphology with regular, homogenous and robust hyphae of a constant diameter and a smooth surface. However, *ΔUvSorA* and *ΔUvSorB* mutants showed considerable alterations, which appeared as a damaged surface with rips and partly squashed. The TEM images of WT mycelia showed a cell wall with uniform layers, defined plasma-membrane and periplasm region with normal thickness. The cytoplasm was clear, and well-organized mitochondria were scattered throughout the cytoplasm. By contrast, the cell walls of Δ*UvSorA* and Δ*UvSorB* mycelia were disrupted and even disappeared in some regions. The plasmalemma appeared faint, undefined and irregular. Discrete cytoplasmic alterations, including a very dense cytoplasm and a mass of disorganized structures, were also observed (Figure 13A). These findings led to our interest in studying the functions of sorbicillinoids in cell wall integrity. Feeding experiments were carried out to understand whether the cell wall integrity can be restored by sorbicillinoids. Different from DMSO controls, when 3 mg/mL of total sorbicillinoids was fed to Δ*UvSorA* or Δ*UvSorB* mutant, the cell wall integrity of the mutants was recovered, the cytoplasm became clear. 50 μg/mL of sorbicillin was also fed to Δ*UvSorA* or Δ*UvSorB* mutant, the cell wall was observed with normal thickness of periplasm region, covered with uniform layers and defined plasma-membrane through TEM (Figure 13B).

## 3. Discussion

### 3.1. Sorbicillinoid Biosythetic Gene Cluster

The *sor* BGCs were predicted in many fungi by antiSMASH database with sorbicillinoids biosynthetic investigation mainly on *Acremonium chrysogenum* [30], *Penicillium chrysogenum* [53], and *Trichoderma reesei* [54]. Genome sequencing revealed that *sor* BGC was existed in *U. virens.* Six genes (i.e., *UvSorA*, *UvSorB*, *UvSorR1*, *UvSorR2*, *UvSorC*, and *UvSorT*) in this cluster were conserved (Figure 1). Through bioinformatic analysis, RNA−seq, qRT−PCR, gene knockout and complementation, and feeding experiments, both *UvSorA* and *UvSorB* in *sor* BGC were confirmed as the PKS genes which were responsible for the synthesis of the basic hexaketide scaffold (i.e., sorbicillin, dihydrosorbicillin) [29]. Monooxygenase (UvSorC) participated in the oxidative dearomatisation of sorbicillin to sorbicillinol. The expression of *sor* BGC was controlled by two transcriptional regulators (UvSorR1 and UvSorR2). UvSorT was the transporter protein, which also participated in the process of sorbicillinoid biosynthesis. UvSorD was a multifunctional flavin-dependent monooxygenase that was required for the production of dimeric sorbicillinoids and other sorbicillinoids (Figure 5). By bioinformatic analysis, *UvSorD* was found excluded in *sor* BGC, and was considered to be located outside of the cluster.

### 3.2. Biological Functions of Sorbicillinoids in Fungi

Secondary metabolites usually have functions in controlling morphological differentiation and biological fitness in fungi. However, the biological functions of sorbicillinoids were seldom studied. It was proposed that their biological functions might be due to their high antioxidant activity that was important for fungi confronted by other organisms, such as plants and higher eukaryotes [55]. In addition, sorbicillinoids could delay overgrowth of *T. reesei*, but they also had strong inhibitory effects on the growth of other fungi [54].

In this study, we confirmed that *UvSorA* and *UvSorB* as the key genes involved in the biosynthesis of sorbicillinoids. To further study the biological functions of sorbicillinoids in *U. virens*, we generated ∆*UvSorA* and ∆*UvSorB* mutants to evaluate their effects on mycelia growth and sporulation. Interestingly, ∆*UvSorA* and ∆*UvSorB* mutants showed a significant increase on mycelia growth and sporulation. When we fed sorbicillinoids to ∆*UvSorA* and ∆*UvSorB* mutants, both mycelia growth rate and spore production level were restored. The mycelial growth seemed negatively correlated with the concentration of sorbicillinoids which was consistent with the previous research [54]. Sorbicillinoids might have function to inhibit overgrowth of *U. virens* and to help *U. virens* maintain territory when facing other fungi [56,57].

Fungal cells were protected by the cell wall. Due to ∆*UvSorA* and ∆*UvSorB* mutants showing increased sensitivities to hyperosmotic, metal cations, cell wall damaging agents and fungicides stresses, suggesting that cell wall integrity was affected. TEM and SEM analysis of hyphae structures showed that the cell walls of Δ*UvSorA* and Δ*UvSorB* mycelia were disrupted and even disappeared in some regions, feeding sorbicillinoids could restore the cell wall integrity. These revealed that sorbicillinoids had protective functions to increase tolerance to cell wall stresses, and therefore increased the environmental competition for *U. virens*.

Sorbicillinoids as the mycotoxins produced by *U. virens* showed strong inhibition against the radicle and germ elongation of rice and lettuce seedlings [16,58]. Both ∆*UvSorA* and ∆*UvSorB* mutants completely lost their ability to synthesize sorbicillinoids. The culture filtrates of the ∆*UvSorA* and ∆*UvSorB* mutants showed a decreased inhibiting on germ elongation of rice seeds (Figure 11). This indicated that sorbicillinoids have a phytotoxic activity on rice seedlings. Further investigation is needed to confirm whether sorbicillinoids acted as the virulence factors in *U. virens*–rice interactions.

Sorbicillinoids had functions in inhibiting mycelial growth and sporulation to prevent overgrowth of *U. virens,* they also maintained cell wall integrity to increase tolerance of *U. virens* in response to abiotic and biotic stresses. However, the detailed mechanisms of these functions of sorbicillinoids are needed for further research.

## 4. Materials and Methods

### 4.1. Fungal Strains, Plasmids and Culture Conditions

The fungal strains and plasmids used in this study are listed in Table S4. The *U. virens* WT strain and its transformants were grown on potato-sucrose agar (PSA) plates (potato, 200 g/L; sucrose, 20 g/L; agar 20 g/L) at 28 °C. Construction and maintenance of plasmids were performed in *Escherichia coli* strain *DH5α* which was cultured in LB medium (tryptone, 10 g/L; yeast extract, 5 g/L; NaCl, 10 g/L) with appropriate antibiotics for plasmid DNA. The vector pCas9-tRp-gRNA and pCBHT were kindly provided by Prof. Jin-Rong Xu (Department of Botany and Plant Pathology, Purdue University, West Lafayette, IN, USA).

### 4.2. Bioinformatic Analysis

The *sor* BGC was predicted using antiSMASH database [32]. Standalone BLAST (version 2.2.28+) was set up on a Microsoft Windows PC system, and BLAST databases were generated for *U. virens* genome (GenBank JHTR00000000.1) and its associated and predicted gene database (both protein and nucleotide sequences). Amino acid sequence of each gene used in this study was downloaded from the National Center for Biotechnology Information (NCBI). The domain was analyzed using CD-search from the NCBI and IBS 1.0 was used to map schematic diagram of the protein domain. Phylogenetic analyses were conducted using the MEGA5.2 (USA, https://www.megasoftware.net/older_versions, accessed on 1 September 2022) [59] with the neighbor-joining algorithm. Protein sequence alignments were performed using ClustalW [60] and ESPript 3.0 [61].

### 4.3. Targeted Gene Deletion and Complementation

To construct the *UvSorA* and *UvSorB* knockout mutants, mycelia of WT strain were harvested from 7-day-old cultures grown in PSA and used for genomic DNA extractions. All primers used in this study are listed in Table S5. For gene replacement via the CRISPR/Cas9 system, the upstream and downstream flanking sequences (~1 kb) of *UvSorA* and *UvSorB* were separately amplified using primers *UvSorA*_5F/5R, *UvSorA*_3F/3R and *UvSorB*_5F/5R, *UvSorB*_3F/3R (Table S5). The resultant PCR products were fused with the geneticin-resistance (Neo^R^) cassette from pFL2 by double-joint PCR [62]. The specific sgRNA for *UvSorA* and *UvSorB* knockout were designed on the website https://portals.broadinstitute.org/gppx/crispick/public (accessed on 20 November 2019). The synthetic sgRNA oligos were annealed and inserted into the BsmBI-digested pCas9-tRp-gRNA. The recombinant plasmids pCas9-tRp-*UvSorA* and pCas9-tRp-*UvSorB* were all confirmed by sequencing. PEG-mediated *U. virens* transformation was performed as described previously [35], with the linear donor DNA fragments and the CRISPR construct pCas9-tRp-*UvSorA* or pCas9-tRp-*UvSorB*. The geneticin-resistance transformants were selected on medium containing 700 μg/mL of G418, and the deletion mutants were verified by PCR amplification. To generate the complementary strains, *UvSorA* and *UvSorB* were amplified with the primers C_*UvSorA*_F/R and C_*UvSorB*_F/R, and then recombined into the vector pCBHT linearized by BamHI and KpnI using EasyGeno Assembly Cloning Kit (Tiangen Biotech, Beijing, China). The resulting constructs, pCBHT-UvSorA and pCBHT-*UvSorB* were transformed into the respective mutant strains via PEG-mediated transformation. The complementary strains were confirmed by PCR amplification. Three of the deletion mutants and three of complemented strains were selected for the experiments of biological functions.

### 4.4. RNA Preparation and qRT−PCR

Gene expression was analyzed at different cultural conditions. *U. virens* was cultivated in GYES medium (yeast extract, 10 g/L; glucose, 10 g/L; starch 10 g/L; NaCl, 5 g/L, CaCO_3_, 3 g/L) and YPD medium (yeast extract, 10 g/L; peptone, 20 g/L; glucose, 20 g/L) for 10 d at 28 °C. Total RNAs from the mycelia in different culture condition strains were extracted by using a TranZol kit (Tiangen Biotech, China). Single strand cDNAs were synthesized using the Fast Quant RT Kit (Tiangen Biotech, China) according to the manufacture’s protocol. The genes coding for putative sorbicillinoid biosynthesis were used for the transcriptional analysis.

Each cDNA sample was performed in triplicate and the average threshold cycle was calculated. Relative expression levels were calculated by using the 2^−ΔΔCT^ method [63]. The β-actin gene served as the internal control for the expression studies. All experiments in this section were performed in three independent biological experiments with three replicates in each test.

### 4.5. Chemical Analysis of Sorbicillinoids in WT Strain, Gene Deletion Mutants, and Complemented Strains of U. virens

For chemical analyses, the WT strain, deletion mutants, and complemented strains were grown on GYES plates at 28 °C for 4 weeks. The same plates with hyphae were extracted with MeOH for three times. The MeOH extracts were combined, and the solvent was removed using a rotatory evaporator under reduced pressure to yield a tawny residue. The residue was dissolved in MeOH and filtered through a microporous filter (pore size, 0.22 μm) for HPLC-DAD and LC-MS analysis. HPLC-DAD analysis of the MeOH extracts was performed on a Shimadzu instrument equipping with SPD-M20A photodiode array detector (LC-20A, Shimadzu Corp., Tokyo, Japan) using an analytic C_18_ column (250 mm × 4.6 mm i.d., 5 μm; Phenomenex Inc., Torrance, CA, USA). The column temperature was set at 30 °C. The mobile phase was composed of methanol (B), and water containing 0.02% TFA (A). A gradient elution program eluting from 60% to 100% MeOH over 40 min was used, and flow rate was 1.0 mL/min. Detection of sorbicillinoids was performed at a wavelength of 370 nm. LC-MS was performed on a Q-TOF-MS 6520 mass spectrometer (Agilent Technologies Santa Clara, CA, USA) coupled to an LC 1260 system with a Phenomenex C18 column (i.d., 150 mm × 2.0 mm, 3 µm) eluting from 30% to 90% acetonitrile over 30 min at 0.25 mL/min. Mass detector operated simultaneously in ESI^-^ mode between 50 and 1400 *m/z*.

### 4.6. Physiology Experiments

The WT strain, deletion mutants, and complemented strains of *U. virens* were cultured in PSB with 160 rpm at 28 °C for 7 days, and the spores were collected to obtain spore suspension with the same concentration (1 × 10^6^ spores/mL) for further studies.

For the growth rate assessment, 1 μL of spore suspension (1 × 10^6^ spores/mL) was inoculated on PSA, GYES, PDA (potato, 200 g/L; glucose, 20 g/L; agar, 20 g/L), YEKM (yeast extract, 10 g/L; glucose, 2 g/L; KH_2_PO_4_, 1.2 g/L; MgSO_4_, 1.2 g/L; agar, 20 g/L), and YPD media and grown at 28 °C for 18 days. The colony diameter measurements and statistical analysis were followed. For sporulation assessment, the spore suspensions were inoculated in PSB and GYES cultures by shaking at 160 rpm and 28 °C for 7 days, spores were collected, and the number of spores were counted by a blood counting plate under a microscope. For the biomass measurement, 1 mL re-suspended spores were cultured in 100 mL of PSB or GYES medium with 160 rpm shaking at 28 °C for 7 days, the hyphae were collected and measured for dry weights.

To test the sensitivity against different stresses, 1 μL of spore suspension (1 × 10^6^ spores/mL) was inoculated in the center of the plate at 28 °C for 18 days. Mycelial growth was assayed after incubation at 28 °C for 18 days on PSA plates and PSA with 0.25 or 0.5 M NaCl; 0.25 or 0.5 M KCl; 0.5 or 1.0 M sorbitol; 2 or 3 mg/mL CR; 0.03% or 0.06% SDS (*w*/*v*); 60 or 120 μg/mL CFW; 5 mM ZnSO_4_; 0.4M MgCl_2_; 0.04M LiCl, 0.08 M MnCl_2_; 0.1 μg/mL epoxiconazole; 0.2 μg/mL difenoconazole; 0.2 μg/mL azoxystrobin; 0.4 μg/mL prochloraz; 0.4 μg/mL carbendazim, respectively. The colony diameter was measured and mycelial growth inhibition in strains was compared with non-treated controls. The inhibition ratio (%) was calculated using the following equation: (average of strain colony diameters on PSA—average of strain colony diameters on PSA with chemical added)/average of strain colony diameters on PSA × 100%. All experiments were repeated three times with three replicates each time.

### 4.7. Feeding Sorbicillinoids in ΔUvSorA and ΔUvSorB Mutants

The crude extract containing sorbicillinoids was obtained from WT strain which was grown on GYES plates at 28 °C for 4 weeks. The same plates with hyphae were extracted with MeOH for three times. The combined MeOH extract was subjected to size-exclusion chromatography over Sephadex LH-20 (CH_2_Cl_2_-MeOH, 1:1, *v*/*v*) to obtain crude sorbicillinoid fraction which contained five main sorbicillinoids (i.e., trichotetronine, dihydrotrichodimer ether A, ustisorbicillinol B, demethyltrichodimerol, and tricho-dimerol) [16]. Crude sorbicillinoid extract was respectively added into Δ*UvSorA* or Δ*UvSorB* mutant at concentrations of 1.0–3.5 mg/mL in distilled water containing 10% DMSO. For comparison purpose, WT, and non-treated Δ*UvSorA* and Δ*UvSorB* mutants were used as the controls. All strains were grown on GYES plates at 28 °C for 21 days. The colony diameter was measured. For sporulation, crude sorbicillinoid fraction (0.5–2.5 mg/mL) were added into Δ*UvSorA* and Δ*UvSorB* mutants, grown in PSB with 160 rpm at 28 °C for 7 days. The concentrations of spores were measured.

### 4.8. Microscopic Observation of Hyphal Morphology

For scanning electron microscope (SEM) observation, hyphal samples were fixed in 2.5% glutaraldehyde buffer overnight at 4 °C. Then, the samples were washed 3 times with 0.1 M sodium phosphate (pH 7.4), 10 min per time. Then, they were washed and dehydrated with a series of ethyl alcohol solutions (30%, 60%, 80%, 90%, 100%). Then, the dried samples were coated with platinum by using a Hitachi E-1045 ion sputter and observed with a scanning electron microscope (SEM, Hitachi S-3400, Tokyo, Japan).

For transmission electron microscope (TEM) observation, the plates adhered with *U. virens* mycelia was cut to the agar plugs about 0.1–0.2 mm thickness after 7 days cultivation. Then the slices were fixed in 2.5% glutaraldehyde for 3 h, washed twice with 0.2 M PBS (pH 7.4) for 0.5 h, post fixed in 1.0% osmium tetra oxide for 2 h, and then washed with PBS again. Samples were dehydrated in a graded ethanol series, passed three times in acetone-ethanol solution, embedded in epoxy at 65 °C for 16 h, and then sectioned into ultrathin pieces with diamond knife, subsequently section staining with uranyl acetate and lead citrate. The micromorphology was observed with the transmission electron microscopy (TEM, JEM-1230, Tokyo, Japan).

### 4.9. Phytotoxic Assays with Culture Filtrates

After 7 days of culture in 100 mL of PDB, the filtrates were collected and centrifuged at 7500 rpm for 6 min. The supernatant was collected and incubated at 75 °C for 40 min. The rice seeds were soaked in 0.1% potassium permanganate with 160 rpm shaking at 28 °C for 50 min and then washed 5 times with sterile distilled water. Fifty seeds were soaked in 25 mL of culture filtrate in a 100 mL–conical flask, and germ elongation was measured after incubation at 28 °C for 7 days.

## 5. Conclusions

In summary, a biosynthetic gene cluster for sorbicillinoids in *U. virens* was identified by employing sorbicillinoids-producing vs. non-producing media, and combining with bioinformatic analysis, RNA-Seq and qRT-PCR. The biological functions of sorbicillinoids were preliminarily revealed by deletion of *UvSorA* and *UvSorB*, complementation, feeding experiments, and phenotypic analysis. The mycelial growth, sporulation and hyphal hydrophobicity were increased for ∆*UvSorA* and ∆*UvSorB* mutants. In addition, the ∆*UvSorA* and ∆*UvSorB* mutants were decreased in tolerance to hyperosmotic, metal cations, cell wall damaging agents and fungicide stresses. Both the microscopic and submicroscopic structures of the deletion mutants were found to be altered. Feeding sorbicillinoids could restore cell wall integrity. Furthermore, culture filtrates of ∆*UvSorA* and ∆*UvSorB* mutants showed a decreased inhibition on germ elongation of germinated rice seeds. The obtained data demonstrated that both *UvSorA* and *UvSorB* were responsible for sorbicillinoid biosynthesis in *U. virens*, and contributed to mycelial growth, sporulation, cell wall integrity, stress responses, and phytotoxic activity through sorbicillinoid mediation. In this study, gene deletion, complementation, and SMs feeding were proved to be effective methods to elucidate biological functions of sorbicillinoids, other strategies such as gene overexpression are also worth studying. It provides a basis for further investigation of biological functions and biosynthesis of sorbicillinoids.

## Data Availability

Not applicable.

## Supplementary Materials

The following supporting information can be downloaded at: https://www.mdpi.com/article/10.3390/ijms231911056/s1. Reference [64] are cited in the Supplementary Materials.
