# Supplementary material for: UvSorA and UvSorB Involved in Sorbicillinoid Biosynthesis Contribute to Fungal Development, Stress Response and Phytotoxicity in Ustilaginoidea virens
Source: Int J Mol Sci. 2022 Sep 21;23(19):11056. doi: 10.3390/ijms231911056 (PMC9570055; doi:10.3390/ijms231911056)
Supplement: Supplementary file 1 [file ijms-23-11056-s001.zip › ijms-1924207-supplementary.pdf]

Supplementary Materials:

UvSorA and UvSorB Involved in Sorbicillinoid Biosynthesis Contribute to Fungal Development, Stress Response and Phytotoxicity in *Ustilaginoidea virens*

Xuping Zhang [1], Dan Xu [1], Xuwen Hou [1], Penglin Wei [2], Jiajin Fu [1], Zhitong Zhao [1], Mingpeng Jing [1], Zhen Shen [1], Daowan Lai [1], Wen-Bing Yin [2,*] and Ligang Zhou [1,*]

[1] State Key Laboratory of Agrobiotechnology, Department of Plant Pathology, College of Plant Protection, China Agricultural University, Beijing 100193, China; zhangxuping5@cau.edu.cn (X.Z.); cauxudan@cau.edu.cn (D.X.); xwhou@cau.edu.cn (X.H.); juajinfu@cau.edu.cn (J.F.); zhitongzhao@cau.edu.cn (Z.Z.); jmp0930@cau.edu.cn (M.J.); zhenshen@cau.edu.cn (Z.S.); dwlai@cau.edu.cn (D.L.)

[2] State Key Laboratory of Mycology, Institute of Microbiology, Chinese Academy of Sciences, Beijing 100101, China; wpl0816@126.com (P.W.)

* Correspondence: yinwb@im.ac.cn (W.-B.Y.); lgzhou@cau.edu.cn (L.Z.)

Contents

Figure S1. Amino acid sequence alignment analysis of UvSorA and UvSorB (*U. virens*), TrSorA and TrSorB (*T. reesei*), PcSorA and PcSorB (*P. chrysogenum*), AcSorA and AcSorB (*A. chrysogenum*), and CgSorA and CgSorB (*C. graminicola*), their functional domains are well conserved. Red highlights indicate the conserved regions of these proteins. (**A**) Part of UvSorA amino acid sequence alignment analysis of enoylreductase (ER) domain. (**B**) Part of UvSorB amino acid sequence alignment analysis of thioesterase/Claisen cyclase (TE/CLC) domain. 2

Figure S2. The biomasses of the tested strains in 100 mL liquid PSB and GYES media. All tested strains were cultured at 160 rpm and 28 °C for 7 days. The mycelia dry weight (g) was measured for statistical analysis. Error bars represent the standard deviation, and asterisk (*) represents a significant difference at $p < 0.05$. At the same culture conditions, Δ*UvSorA* and Δ*UvSorB* mutants showed higher dry weight accumulation than WT and complemented strains. 3

Table S1. Nineteen biosynthetic gene clusters (BGCs) for secondary metabolites (SMs) were predicted in *Ustilaginoidiea virens* using antiSMASH 6.0.1. 4

Table S2. The genes and proteins (enzymes) predicted through deep bioinformatic annotation of *sor* BGC in different fungi. 5

Table S3. The DEGs in *sor* BGC of *U. virens* cultured in sorbicillinoid producing medium vs non-producing medium (GYES/YPD). 6

Table S4. Fungal strains and plasmids used in this study. 7

Table S5. The primers used in this study. 8

Supplementary References 9

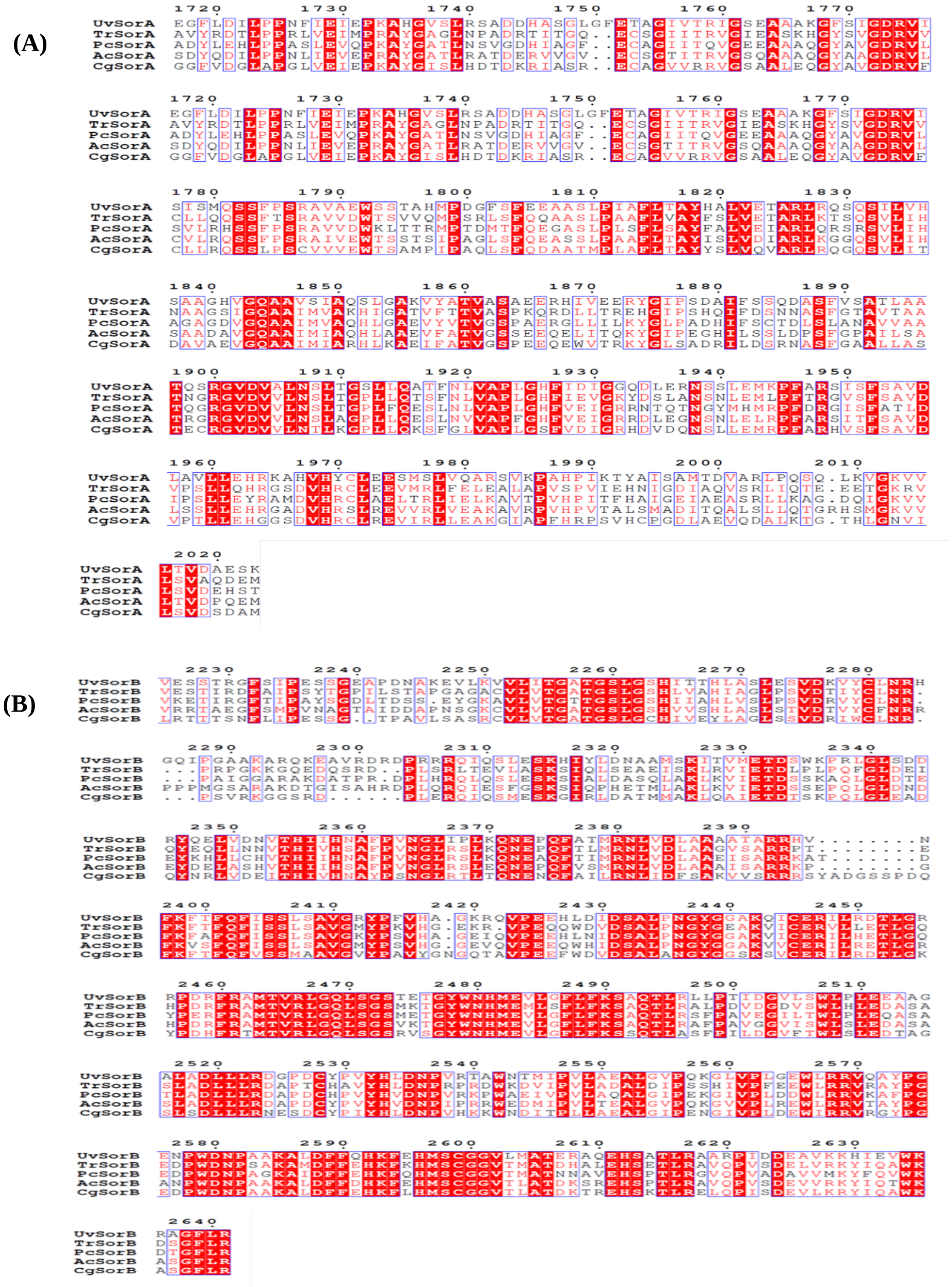

Figure S1. Amino acid sequence alignment analysis of UvSorA and UvSorB (*U. virens*), TrSorA and TrSorB (*T. reesei*), PcSorA and PcSorB (*P. chrysogenum*), AcSorA and AcSorB (*A. chrysogenum*), and CgSorA and CgSorB (*C. graminicola*), their functional domains are well conserved. Red highlights indicate the conserved regions of these proteins. (**A**) Part of UvSorA amino acid sequence alignment analysis of enoylreductase (ER) domain. (**B**) Part of UvSorB amino acid sequence alignment analysis of thioesterase/Claisen cyclase (TE/CLC) domain.

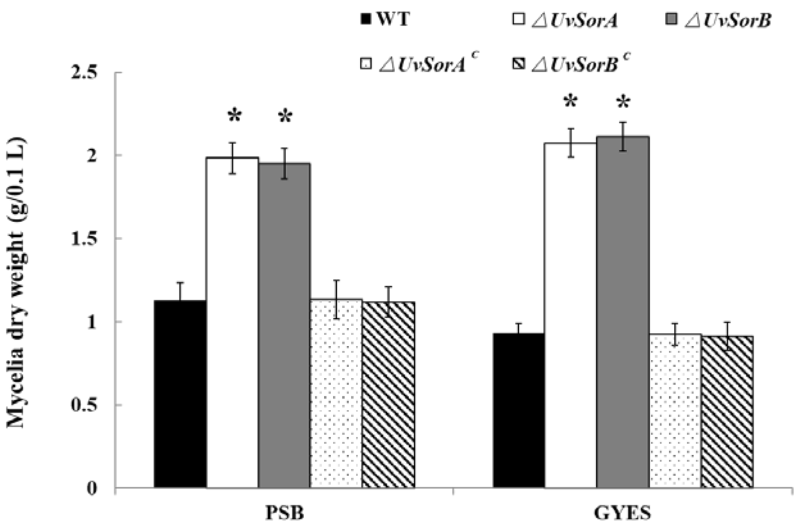

Figure S2. The biomasses of the tested strains in 100 mL liquid PSB and GYES media. All tested strains were cultured at 160 rpm and 28 °C for 7 days. The mycelia dry weight (g) was measured for statistical analysis. Error bars represent the standard deviation, and the asterisk (*) represents a significant difference at $p < 0.05$. At the same culture conditions, Δ*UvSorA* and Δ*UvSorB* mutants showed higher dry weight accumulation than WT and complemented strains.

Table S1. Nineteen biosynthetic gene clusters (BGCs) for secondary metabolites (SMs) were predicted in *Ustilaginoidiea virens* using antiSMASH 6.0.1.

BGC Cluster	Type	From	To	Most Similar Known Cluster	Similarity (%)
1.1	NRPS	3,030,817	3,079,764	-	-
1.2	T1PKS	5,125,293	5171.437	-	-
1.3	Terpene	7,157,430	7,179,071	-	-
1.4	T1PKS	7,220,622	7,269,047	Ustilaginoidins	100
2.1	NRPS	1,987,709	2,029,676	-	-
2.2	T1PKS	2,113,814	2,157,754	-	-
2.3	T1PKS	6,752,222	6,809,199	Sorbicillinoids	71
3.1	NRPS-like	434,033	474,464	-	-
3.2	NRPS-like	3,797,030	3,841,215	-	-
4.1	Terpene	431,004	451,915	-	-
4.2	NRPS	731,621	783,641	-	-
4.3	NRPS	2,398,342	2,443,699	Dimethylcoprogen	100
4.4	NAPAA	4,030,011	4,064,012	-	-
4.5	NRPS-like	5,320,383	5,363,774	-	-
5.1	Terpene	257,703	280,014	Gibberellin	28
6.1	Terpene	2,807,580	2,829,037	-	-
6.2	NAPAA	3,259,797	3,293,699	-	-
6.3	T1PKS	3,317,702	3,365,048	-	-
7.1	NRPS-like	230,418	272,198	-	-

Note: NRPS, non-ribosomal peptide synthetase cluster; T1PKS, type I polyketide synthase cluster; NAPAA, non-α- poly-amino acids like ε-poly-lysine.

Table S2. The genes and proteins (enzymes) predicted through deep bioinformatic annotation of *sor* BGC in different fungi.

**Protein of *sor* BGC in *U. virens***	**Corresponding Protein in other Fungi. Coverage/Identity values (%) of *U. virens* ORFs to Those of other Fungi are Given in the Parentheses**			**Putative Function**
	Penicillium chrysogenum	*Trichoderma reesei*	*Acremonium chrysogenum*	
UvSorR1 (UV8b_6009)	Pc21g05050 (97/49)	Triredraft_102499 (99/44)	ACRE_048150 (98/57)	Fungal specific transcription factor domain
UvSorB (UV8b_6010)	Pc21g05070 (99/65)	Triredraft_73621 (99/64)	ACRE_048170 (100/65)	Polyketide synthase
UvSorA (UV8b_6011)	Pc21g05080 (99/70)	Triredraft_73618 (99/72)	ACRE_048180 (99/71)	Polyketide synthase
UvSorR2 (UV8b_6012)	Pc21g05090 (94/56)	Triredraft_102497 (97/55)	ACRE_048120 (92/55)	Fungal specific transcription factor
UvSorT (UV8b_6013)	Pc21g05100 (99/77)	Triredraft_43701 (95/74)	ACRE_048130 (99/77)	MFS multidrug transporter
UV8b_6014	Pc22g18490 (86/44)	Triredraft_80733 (87/53)	-	Hypothetical protein
UV8b_6015	-	-	-	Glyoxalase bleomycin resistance protein
UV8b_6016	Pc21g09730 (84/51)	Triredraft_3055 (77/66)	ACRE_019070 (82/59)	3-Oxoacyl-(acyl-carrier-protein) reductase
UvSorC (UV8b_6017)	Pc21g05060 (99/58)	Triredraft_73623 (98/58)	ACRE_048160 (98/62)	Salicylate 1-monooxygenase
UV8b_6018	-	-	-	Hypothetical protein

Note: Percentage coverage/identify values of *Ustilaginoidea virens* ORFs to those of *Penicillium chrysogenum* DS68530, *Trichoderma reesei* QM6a, *Acremonium chrysogenum* ATCC 11550, are given in parentheses.

Table S3. The DEGs in *sor* BGC of *U. virens* cultured in sorbicillinoid producing medium vs non-producing medium (GYES/YPD).

**Gene ID**	**log_2FC** (GYES/YPD)	***P* value**	**Regulated**	**Description**
UV8b_6009	2.77382	2.41E-05	up	Fungal specific transcription factor domain
UV8b_6010	4.43852	2.03E-05	up	NR-polyketide synthase
UV8b_6011	2.88214	1.15E-05	up	HR-polyketide synthase
UV8b_6012	2.21301	1.27E-06	up	Fungal specific transcription factor
UV8b_6013	2.70416	3.24E-05	up	MFS multidrug transporter
UV8b_6014	-1.52964	0.0288	down	Hypothetical protein
UV8b_6015	-1.31014	0.0111	down	Glyoxalase bleomycin resistance protein
UV8b_6016	-0.54496	0.0051	down	Dioxygenase superfamily
UV8b_6017	4.76262	2.84E-10	up	3-Oxoacyl-(acyl-carrier-protein) reductase
UV8b_6018	-1.06986	0.0015	down	Salicylate 1-monooxygenase

Note: This table is supplemental to the research results.

Table S4. Fungal strains and plasmids used in this study.

Fungal Strain/Plasmid		Description	Reference
**Fungal strain**	*Ustilaginoidea virens* P1	Wild-type	[1]
	Δ*UvSorA*	*UvSorA* deletion mutant of P1	This study
	Δ*UvSorB*	*UvSorB* deletion mutant of P1	This study
	Δ*UvSorA*^{C}	*UvSorA* complement strains mutant of *ΔUvSorA*	This study
	Δ*UvSorB*^{C}	*UvSorB* complement strains mutant of *ΔUvSorB*	This study
**Plasmid**	pCas9-tRp-gRNA	Cas9-gRNA vector with the tRNA promoter	[2]
	pCBHT	The complemented vector	[1]
	pFL2	pFL2 containing geneticin-resistance (Neo^R)	[3]
	pCas9-tRp-UvSorA	Cas9-gRNA vector with the UvSorA spacer	This study
	pCas9-tRp-UvSorB	Cas9-gRNA vector with the UvSorB spacer	This study
	pCBHT-UvSorA	The vector pCBHT containing UvSorA	This study
	pCBHT-UvSorB	The vector pCBHT containing UvSorB	This study

Note: This table is supplemental to the experimental procedure.

Table S5. The primers used in this study.

Primer Name	Oligonucleotide Sequence (5′-3′)	Function
UvSorA_5F	GCTTCACGGTAAGATGCGGA	UvSorA′5 flanks amplification
UvSorA_5R	cagatacggcagagaaatcgcaacctcTTGCTTCCACAAAGTCGTCG	
UvSorA_3F	gtttagattccaagtgtctactgctggcGTCTCACAAAGTTGGCGGC	UvSorA′3 flanks amplification
UvSorA_3R	CTCCAAACCATCAGTCGCAAC	
UvSorA _F	ACCGAAAACACAACATGGCG	UvSorA transformant screening
UvSorA _R	CGAATGAGGCGTCTTGACTG	
KOUvSorA_5F	GATGACTACGAGCGGTCCCA	UvSorA transformants 5F and 3F screening
KOUvSorA_3R	CCTACAGCGGTATTGAACTCG	
UvSorA_spacer_F	acctGTAGTGGTGCTACAACTCGT	Constructing pCas9-tRp-UvSorA vector
UvSorA_spacer_R	aaacACGAGTTGTAGCACCACTAC	
C_UvSorA_F	agaaatcgcaacctcgaattcTCGGAATCTCCTCGCACAG	UvSorA amplification
C_UvSorA_R	gttgagtggaatgatggatccCTATGCCGCCACAAACTCAA	
UvSorB_5F	GAAGGACGATTATGCCGTCG	UvSorB′5 flanks amplification
UvSorB_5R	cagatacggcagagaaatcgcaacctcCCAGCAGCAATGGTCAAGTG	
UvSorB_3F	gtttagattccaagtgtctactgctggcTGATGAGGCGGTGAAGAAGC	UvSorB′3 flanks amplification
UvSorB_3R	CTGCCTCGTAGGGCTGTAAC	
UvSorB_F	GGAGATGATGGGAACCTTGC	UvSorB transformant screening
UvSorB_R	CGTCAATAGTCGGCAACAAGC	
KOUvSorB_5F	AATGGCGATTGGCTCTGTAG	UvSorB transformants 5F and 3F screening
KOUvSorB_3R	GCGAGGCTTATGTCAACTCG	
UvSorB_spacer_F	acctTTATGACTACAAGAGCCCCC	Constructing pCas9-tRp-UvSorB vector
UvSorB_spacer_R	aaacGGGGGCTCTTGTAGTCATAA	
C_UvSorB_F	agaaatcgcaacctcgaattcCGCTCATTGTTTGGGTGGTA	UvSorB amplification
C_UvSorB_R	gttgagtggaatgatggatccCTACCGCAAAAATCCAGCC	
GEN_F	GAGGTTGCGATTTCTCTGCCGTATCTG	GenR amplication from pFL2
GEN_R	GCCAGCAGTAGACACTTGGAATCTAAAC	

855_R	TGTTGGGTTTGAGCTAGGTGGG	Upstream and downstream flanks amplification
856_F	GAATGGTCAAATCAAACTGCTAGATAT	
852_F	TCGGCTATGACTGGGCACAACA	Tansformants GenR screening
850_R	GAGCGGCGATACCGTAAAGCAC	
*β-actin*_F	CCGTGAGAAGATGACCCAGA	*U.virens β-actin* as the control
*β-actin*_R	GGCGAAACCCTCGTAGATGG	
RT_UvSorA_F	GCACAAAGACGCCAGTGAAG	qRT-PCR for UvSorA
RT_UvSorA_R	GCGGTCGACACAGGAAGATT	
RT_UvSorB_F	GGACCTCCATGTGGTTCCAG	qRT-PCR for UvSorB
RT_UvSorB_R	TGATAGCGCTCTTCCCAACG	
RT_UvSorC_F	CGTCTCTGTCCACGTCCTTG	qRT-PCR for UvSorC
RT_UvSorC_R	CAGAGGCGTTGGCTGTTTTG	
RT_UvSorR1_F	CAGGGCCTCCACCAAATTCT	qRT-PCR for UvSorR1
RT_UvSorR1_R	AGGTGGCTGTTCTCGTCAAG	
RT_UvSorR2_F	GCGGTCGACACAGGAAGATT	qRT-PCR for UvSorR2
RT_UvSorR2_R	CGCTGTGGCAGTGCAATTAG	
RT_UV_6014_F	GTTCCTGACCCGTCTGTCTC	qRT-PCR for UV8b_6014
RT_UV_6014_R	TCAGATCGTGGCTCCCCTTA	
RT_UV_6015_F	AACTCAGTAGCGCCATTCCC	qRT-PCR for UV8b_6015
RT_UV_6015_R	AAAACCCCAGCGATGCTTGT	
RT_UV_6016_F	AAAGAGCCCAACAAGAGTGGA	qRT-PCR for UV8b_6016
RT_UV_6016_R	GTGTGGTTTTCCTGGCATCG	
RT_UV_6018_F	ACTTGTGAACCCAGACGAGC	qRT-PCR for UV8b_6018
RT_UV_6018_R	TCGTGTTTCGGCGTACATCT	

Note: This table is supplemental to the experimental procedure.

Supplementary References

1. Zheng, D.; Wang, Y.; Han, Y.; Xu, J.-R.; Wang, C. *HvHOG1* is important for hyphal growth and stress responses in the rice false smut fungus *Ustilaginoidea virens*. *Sci. Rep.* **2016**, *6*, 24824.
2. Liang, Y.; Han, Y.; Wang, C.; Jiang, C.; Xu, J.-R. Targeted deletion of the *USTA* and*UvSLT2* genes efficiently in *Ustilaginoidea virens* with the CRISPR-Cas9 system. *Front. Plant Sci.* **2018**, *9*, 699.
3. Zhou, X.; Li, G.; Xu, J.- R. Efficient approaches for generating GFP fusion and epitope-tagging constructs in filamentous fungi. *Methods Mol. Biol.* **2011**, *722*, 199–212.
